# Physical examination skills training: Faculty staff vs. patient instructor feedback—A controlled trial

**DOI:** 10.1371/journal.pone.0180308

**Published:** 2017-07-10

**Authors:** Markus Krautter, Katja Diefenbacher, Jobst-Hendrik Schultz, Imad Maatouk, Anne Herrmann-Werner, Nadja Koehl-Hackert, Wolfgang Herzog, Christoph Nikendei

**Affiliations:** 1 Department of Nephrology, University of Heidelberg, Heidelberg, Germany; 2 Department of General Internal Medicine and Psychosomatics, University of Heidelberg, Medical Centre, Heidelberg, Germany; 3 Department of Psychosomatic Medicine and Psychotherapy, University of Tübingen, Tübingen, Germany; 4 Department of General Practice and Health Services Research, University of Heidelberg, Heidelberg, Germany; Robert Bosch Krankenhaus, GERMANY

## Abstract

**Background:**

Standardized patients are widely used in training of medical students, both in teaching and assessment. They also frequently lead complete training sessions delivering physical examination skills without the aid of faculty teaching staff–acting as “patient instructors” (PIs). An important part of this training is their ability to provide detailed structured feedback to students which has a strong impact on their learning success. Yet, to date no study has assessed the quality of physical examination related feedback by PIs. Therefore, we conducted a randomized controlled study comparing feedback of PIs and faculty staff following a physical examination assessed by students and video assessors.

**Methods:**

14 PIs and 14 different faculty staff physicians both delivered feedback to 40 medical students that had performed a physical examination on the respective PI while the physicians observed the performance. The physical examination was rated by two independent video assessors to provide an objective performance standard (gold standard). Feedback of PI and physicians was content analyzed by two different independent video assessors based on a provided checklist and compared to the performance standard. Feedback of PIs and physicians was also rated by medical students and video assessors using a questionnaire consisting of 12 items.

**Results:**

There was no statistical significant difference concerning overall matching of physician or PI feedback with gold standard ratings by video assessment (p = .219). There was also no statistical difference when focusing only on items that were classified as major key steps (p = .802), mistakes or parts that were left out during physical examination (p = .219) or mistakes in communication items (p = .517). The feedback of physicians was significantly better rated than PI feedback both by students (p = .043) as well as by video assessors (p = .034).

**Conclusions:**

In summary, our study demonstrates that trained PIs are able to provide feedback of equal quantitative value to that of faculty staff physicians with regard to a physical examination performed on them. However, both the students and the video raters judged the quality of the feedback given by the physicians to be significantly better than that of the PIs.

## Introduction

The physical examination of a patient is an essential clinical competence of physicians, and along with comprehensive history taking, flags the beginning of primary patient-doctor relationships. Moreover, history taking and physical examination form the basis for establishing a diagnosis, planning further diagnostic steps, and developing a therapeutic scheme for the patient’s care. Accordingly, the acquisition of physical examination skills constitutes a centerpiece of medical education [1]. However, several studies indicate severe shortcomings in students’ physical examination competencies [2,3]. In a recent study, we revealed significant deficits in the ability of final-year medical students to perform a detailed physical examination of standardized patients [4], with only 63% of correctly performed procedural steps. However, the manner in which physical examination skills should be delivered is still subject to discussion [5–7].

Standardized Patients (SPs) are specially trained laypersons who present learned symptoms or diseases in a standardized, non-varying manner for didactic purposes. The assignment of SPs, both in teaching and assessment, has a long tradition in medical education [8–14]. SPs have also been used successfully for the teaching of physical examinations [15–19] − mostly by assisting a faculty staff trainer − and for the assessment of examination skills via objective structured clinical examinations (OSCE) [15,20]. In some studies, SPs have even led a complete training session, sometimes being termed “patient instructors” (PIs), who deliver physical examination skills without the aid of faculty teaching staff [15,21].

A major didactic element in medical education in general, and in the assignment of SPs in particular, is seen in the delivery of professional, structured feedback, which has been shown to exert an enduring effect when training medical students and physicians [22–27]. SPs undergo extensive feedback training prior to their deployment in student teaching [28–31]. In terms of the delivery and feedback related to physical examination skills, PIs have to take into account not only the quality of communication, but also the correctness of medical procedures, often without having a medical professional background. To the best of our knowledge, no study to date has assessed the quality of physical examination-related feedback by PIs. Specifically, randomized controlled studies comparing PI feedback to faculty staff feedback following a physical examination are completely lacking.

The present study therefore aimed to evaluate the following hypotheses: Compared to physician feedback, PI feedback is not inferior in terms of a) quantitative measures, in terms of the number of named feedback items observed by objective video-assessor ratings and b) qualitative measures, reflected in questionnaire ratings both by students and by video assessors.

## Material and methods

### Trial design

The study prospectively investigated the quality of feedback given by PIs vs. faculty staff physicians–in terms of congruence with gold-standard ratings and questionnaire ratings. For this purpose, we created physical examination small-group teaching settings, each comprising one PI, one faculty staff member and one medical student. In total, fourteen different PIs and 14 different faculty staff physicians delivered feedback to 40 medical students (two or three medical students per PI and faculty staff member). First, the medical student performed a physical examination of the respective PI while the physician observed his/her performance. Second, the student received feedback from both the PI and the physician, independently from each other. The feedback of the PI and the physician was then content-analyzed by two different independent video assessors based on a provided checklist. Finally, the results were compared to an objective performance standard as previously described elsewhere [4,32,33].

### Participants

#### Patient instructors sample

Patient instructors (n = 14; 4 female, 9 male; mean age 41.1 ±15.3years) all had considerable experience in communication skills training and feedback prior to the physical examination training (mean time of serving as an SP 3.4 ±2.7 years; mean number of roles 10.3 ±13.3). PIs were informed that the purpose of the study was to evaluate the final-year students’ physical examination skills and were otherwise blinded to the study design.

#### Physicians sample

Physicians (n = 14; 4 female, 9 male; mean age 33.6 ±6.0years) were experienced internal medicine residents, who had substantial experience in teaching and supervising physical examination (mean work experience in internal medicine 2.9 ±2.2 years, mean teaching experience 4 ±4.0 years). In line with the instruction of the PIs, physicians were also told that the purpose of the study was to evaluate the final-year students’ physical examination skills and were otherwise blinded to the study design.

#### Student sample

A total of 40 (25 female, 15 male; mean age 24.8 ±1.4 years) final-year students agreed to participate in the study. The students were not told that the study aimed to directly compare feedback skills, and were instead informed that the purpose of the study was to investigate their physical examination skills and that they would receive feedback from both the SP and the physician.

### Acquisition of data

The trial was conducted over a three-week period at the beginning of the winter semester at the University of Heidelberg, Germany. Data were collected on the premises of the Department of Internal and Psychosomatic Medicine at Heidelberg University Hospital.

### Ethics

Ethical approval was granted by the ethics committee of the University of Heidelberg (Nr. S-009/2015). Written consent was obtained from all participants. Study participation was voluntary and all candidates were assured of anonymity and confidentiality.

### Patient instructor physical examination training

All PIs underwent two four-hour-long physical examination training sessions. The training began with instruction on basic anatomical and physiological features of the cardiovascular system, the lungs, the abdomen and the thyroid gland, followed by instruction on physical examination skills. By the end of the training, the PIs were therefore themselves able to perform a physical examination of the respective organ systems. Additionally, the training also focused on the correct recognition of physical examination skills (e.g. the correct placement of the stethoscope during auscultation or the correct depth of palpation during abdominal examination). Finally, PIs were trained in giving specific feedback on physical examination skills, including accompanying communication skills.

#### Physical examination feedback session

**Fig 1:**
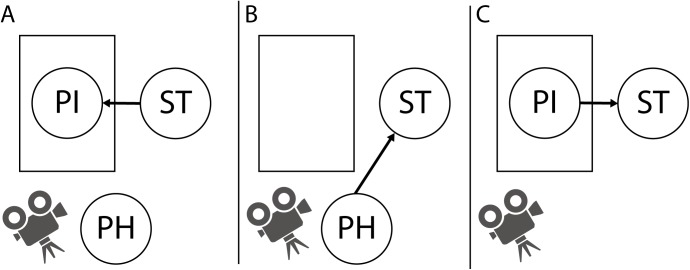
A) Student (ST) performs a physical examination of a patient instructor (PI), while the physician (PH) watches. Student then receives feedback from either physician (B) or PI first (C) while the other one leaves the room.

All 14 PIs were randomly assigned to one of the 14 physicians, and the final-year medical students were randomly assigned to these 14 PI-physician dyads. Twelve dyads performed three sessions, while two dyads performed two sessions, resulting in 40 sessions with 40 student participants overall. The students were given role-playing instructions asking them to perform a pre-employment medical check-up of a PI, including a detailed physical examination of the cardiovascular system, the lungs, the abdomen and the thyroid gland [4]. In line with normal teaching situation procedure, during the examination of the respective PI, the physician was able to make notes on mistakes made by the student on a provided checklist. To avoid interrupting the procedure, the PI had to remember any noticeable problems but was able to make notes afterwards. Following the examination, both the PI and the physician gave feedback to the student separately while the other one left the room (Fig 1). The PI gave feedback first in half of the cases, and the physician gave feedback first in the other half. To enable a subsequent comparison of the feedback, PIs and physicians were asked to give complete feedback, including all mistakes or omitted parts of the physical examination as well as things that were performed well.

### Comparative assessment of standardized patient and physician feedback

First, medical students’ physical examination performances were video-recorded and subsequently rated by two independent video assessors using binary checklists [34] (“item performed correctly”, “item not performed/not performed correctly”) in line with the University-wide physical examination standards. This enabled the development of a gold-standard rating against which PI and medical staff feedback could be compared [4,32]. To obtain a more differentiated pattern, items were categorized into “major” procedural key steps, which are indispensable for a high standard of physical examination, and “minor” procedural steps, which contribute to a more detailed physical examination. Simultaneously, feedback of PIs and physicians was content-analyzed by two different independent video assessors based on the same checklist (“item mentioned was performed correctly”, “item mentioned was not performed or performed incorrectly”, “item was not mentioned”). The resulting ratings were than compared to the gold standard.

Feedback of PIs and physicians was also rated by medical students using a questionnaire consisting of 12 items referring to content (7 items), communication (2 items), and quality (3 items) of the provided feedback. The same questionnaire was used by independent video raters to assess quality of feedback.

#### Video raters

The video raters (one female, one male, 35.0 ±1.0 years) were experienced internal medicine residents who had experience in training and rating students’ physical examination skills.

### Statistical analysis

Data are presented as means ± standard deviation (SD). Non-parametric Mann-Whitney U tests were used for ordinal data. Distribution of group characteristics was compared by Chi-square tests. All data are presented as absolute numbers and percentages or mean and standard deviation. To calculate differences between objective video ratings, the rating scores for PI and physician feedback were aggregated for all of the students assessed by a single teacher or PI (nested design). Ratings from the two video raters were combined into one single rating. A p-value <0.05 was considered to be statistically significant. Standardized inter-rater reliability for the two video assessors was calculated based on intraclass correlation coefficient (ICC) type C. Raw data were processed using Microsoft EXCEL. The software package STATISTICA (Statsoft, Inc, Tulsa, OK, USA) was used for statistical analysis.

## Results

### Objective quantitative comparison of PI and physician feedback by video raters

There was no statistically significant difference concerning overall matching of physician or PI feedback with gold-standard ratings by video assessment (p = .219). There was also no statistically significant difference when focusing only on items that were classified as major key steps (p = .802), mistakes or parts that were left out during physical examination (p = .219) or mistakes in communication items (p = .517; see Table 1).

**Table 1:** Objective comparison of physician and PI feedback with gold standard. Values are percentage of matching with gold-standard checklist ratings.

	Physician (%)	% items	ICC^5^	PI (%)	% items	ICC	p^6^
Overall matching of feedback with gold standard	86.79	26.33^1^	0.697	89.83	22.19^1^	0.846	.219
Matching with gold standard concerning key steps by students	75.95	34.20^2^	0.684	76.73	26.79^2^	0.908	.802
Matching with gold standard concerning mistakes by students	86.79	27.00^3^	0.697	89.83	25.31^3^	0.846	.219
Matching with gold standard concerning communication by students	71.48	15.45^4^	0.493	73.64	11.48^3^	0.058	.517

^1^Percentage of items that received feedback based on all 147 items.

^2^Percentage of items that received feedback based on 53 key step items.

^3^Percentage of items that received feedback based on students’ mistakes (individual number of items for every student).

^4^Percentage of items that received feedback based on 61 communication items.

^5^Intraclass correlation coefficient (ICC) type C.

^6^t-test for independent samples

### Questionnaire assessment of PI and physician feedback by video assessors

Concerning overall ratings by video assessors, physician feedback was significantly better rated than PI feedback (p = .043). There was no statistically significant difference concerning four items (see Table 2).

**Table 2:** Qualitative comparison of PI (n = 14) and physician (n = 14) feedback by video assessors.

Items	PI (n = 14)		Physician (n = 14)		p-value
	Mean	(SD)	Mean	SD	
…gave detailed feedback on the execution of the physical examination (PE).	3.71 (1.25)		2.57 (1.34)		<0.001
…gave detailed feedback on the compliance with framework conditions and on the sequence of the performed PE.	3.66 (1.17)		2.82 (1.27)		0.001
…gave constructive feedback regarding the performance of the physical examination of the thyroid gland	3.63 (1.06)		2.75 (1.16)		<0.001
…gave constructive feedback regarding the performance of the physical examination of the heart.	3.64 (1.20)		2.67 (1.11)		<0.001
…gave constructive feedback regarding the performance of the physical examination of the lungs.	3.79 (1.21)		2.79 (1.25)		<0.001
…gave constructive feedback regarding the performance of the physical examination of the abdomen.	3.78 (1.19)		2.78 (1.23)		<0.001
…gave constructive feedback regarding the doctor-patient communication during the PE.	3.05 (1.20)		3.21 (1.32)		0.500
…was attentive and focused while giving feedback.	2.19 (0.56)		2.05 (0.85)		0.456
…was friendly while giving feedback.	1.96 (0.37)		1.91 (0.51)		0.504
…gave specific examples while giving feedback.	2.41 (0.70)		2.13 (0.74)		0.038
…seemed competent in the field of PE while giving feedback.	3.19 (1.28)		1.91 (0.65)		<0.001
…seemed well prepared with regard to giving feedback (sandwich technique; feedback rules).	3.11 (1.45)		2.57 (1.22)		0.033
Overall	3.18 (1.05)		2.51 (1.05)		0.043

Values are shown as mean and standard deviation. Likert-scale ratings ranging from 1 (I fully agree) to 6 (I completely disagree)

### Questionnaire assessment of PI and physician feedback by students

Concerning overall ratings by students, physician feedback was significantly better rated than PI feedback (p = .034). There was no statistically significant difference concerning five items (see Table 3).

**Table 3:** Qualitative comparison of PI (n = 14) and physician (n = 14) feedback by students.

Items	PI (n = 14)		Physician (n = 14)		p-value
	Mean	(SD)	Mean	SD	
…gave detailed feedback on the execution of the physical examination (PE).	1.83 (1.03)		1,28 (0.55)		0.006
…gave detailed feedback on the compliance with framework conditions and on the sequence of the performed PE.	1.75 (0.84)		1,50 (0.78)		0.126
…gave constructive feedback regarding the performance of the physical examination of the thyroid gland	1.83 (1.08)		1,28 (0.68)		0.004
…gave constructive feedback regarding the performance of the physical examination of the heart.	1.80 (0.91)		1,25 (0.54)		0.001
…gave constructive feedback regarding the performance of the physical examination of the lungs.	1.98 (1.10)		1,40 (0.84)		0.005
…gave constructive feedback regarding the performance of the physical examination of the abdomen.	1.95 (1.13)		1,38 (0.59)		0.014
…gave constructive feedback regarding the doctor-patient communication during the PE.	1.33 (0.57)		1,30 (0.56)		0.812
…was attentive and focused while giving feedback.	1.03 0.16)		1,20 (0.56)		0.133
…was friendly while giving feedback.	1.10 (0.30		1,10 (0.30)		1.00
…gave specific examples while giving feedback.	1.36 (0.63)		1,30 (0.52)		0.836
…seemed competent in the field of PE while giving feedback.	2.25 (1.50)		1,21 (0.47)		0.001
…seemed well prepared with regard to giving feedback (sandwich technique; feedback rules).	1.73 (1.09)		1,35 (0.74)		0.092
Overall	1.66 (0.86)		1.30 (0.59)		0.034

Values are shown as mean and standard deviation. Likert-scale ratings ranging from 1 (I fully agree) to 6 (I completely disagree)

### Interrater reliability of feedback ratings

Interrater reliability of PI and physician feedback ratings (Table 2) was satisfactory to good (0.52 and 0.58, respectively).

## Discussion

The present study prospectively examined whether feedback provided by specially trained PIs regarding a physical examination carried out on them is comparable with feedback provided by faculty staff physicians observing the examination. To this aim, both the examination procedure conducted by the student and the feedback from the PI and the physician were video-recorded. In a first step, the examination was evaluated by video raters using binary checklists, which subsequently served as gold standard. The feedback was then compared with this checklist in order to quantify the points mentioned in the feedback. Overall, both the physician feedback and the PI feedback contained only a small percentage of the possible items (between 11 and 34%). In terms of matching with the gold standard, no significant difference in performance was found between PIs and physicians. As the 147-item checklist is very comprehensive, it is understandably not possible to list all of these points individually in one feedback session. Therefore, an examination of the pure mistakes, the key steps and the communicative aspects was conducted. Again, no significant differences emerged between the PI and physician feedback on any of these points.

This equally good result of faculty staff physicians and PIs is particularly striking as the PIs were unable to make any notes during the examination, and had to remember the individual points, while the physicians–as is customary in normal teaching–already made notes for later feedback during the examination. Moreover, the finding shows that with corresponding training [35], it is possible for PIs to become very well qualified in a very short time.

However, the video raters evaluated the quality of the physician feedback as significantly better overall than that of the PI feedback. When observing the individual items, it becomes apparent that this difference is primarily–though not exclusively–based on the better feedback regarding the technical implementation of the physical examination. This indicates that despite intensive training, the PIs have not yet been able to acquire sufficient routine in this field, and possibly did not make as confident an impression on the students and video raters, even though no objective difference in the feedback on the items was apparent. As these were the first assignments of the trained PIs, this aspect might improve in the future with greater experience and more assignments. The subjective evaluation by the students also showed higher ratings for the physicians than for the PIs, although it should be noted that both groups received high ratings.

To our knowledge, the present study is the first to provide insight into the quality of PI feedback in direct comparison with physician feedback in relation to a complex and extensive task like the physical examination of four organ systems. The use of SPs as PIs in the area of physical examination is nothing new, having already been described some 20 years ago [36,37], although in most cases, no feedback on the examination steps is given. An exception to this is the use of PIs within OSCEs: In a survey of all German-speaking universities, it was found that in 31 of 39 participating universities, SPs were used for the provision of feedback, and in five universities they were also used as raters within the framework of OSCEs [38]. In this context, SPs frequently do not assess the examination per se, but rather assess, for example, whether the student deals with the SP in a professional manner [39]. Moreover, in the case of OSCEs, the extent of the task is also smaller than in our study, and the checklists accordingly shorter. Another finding that has not been previously described is the overall low feedback rate of both the physicians and the PIs. Presumably, this arises from the feedback provider’s need to primarily give feedback on the most salient positive and negative points, so that the student is not overwhelmed by a large amount of items. Nevertheless, it should be noted that only around 30% of key steps received feedback.

Future studies should employ different examinations to investigate how feedback providers select the items on which to give feedback, and the extent to which this proportion of feedback increases in situations encompassing fewer items to be rated. On the whole, the area of feedback provision by SPs remains relatively unexplored, with the AMEE Guide 2009 already concluding that “There is also a clear lack of studies with regard to the training for and the effect of giving feedback by SPs” [29]. However, the studies published since then have been devoted to other situations: For instance, in their study, Bowman et al studied the feedback of 8 licensed physical therapists serving as standardized patients for practical examinations in comparison to a course instructor [40]. Based on the intraclass correlation coefficient there was a significant difference between scores so the authors concluded, that standardized patients might not be an adequate replacement for an instructor. However, the results of the standardized patients and the instructor were not compared to a gold standard. May et al. describe the use of PIs within a Clinical Performance Examination (CPX) Test, in which both history-taking and physical examination as well as patient consultation are assessed [41]. Through intensive training, a checklist congruence of >85% was reached, which was also achieved in our study, at least in terms of matching with the gold standard. However, May et al. do not describe the total extent of the checklist and the proportion of physical examination. Nevertheless, it can be assumed that the number of assessed items is a great deal higher in the present study.

## Limitations

Several limitations of this study should be mentioned. First, the percentage of points that received feedback is relatively low compared to the gold standard. This does, however, reflect everyday life, in which feedback must be limited to the points that appear to be most important, and it is almost never possible to provide comprehensive feedback. Although our PIs had completed extensive training, these were their first “real” assignments as a PI who is examined and provides feedback directly afterwards, while the physicians already had several years of experience both in physical examination and in teaching itself. Therefore, it may be the case that with the experience that the PIs will gather over time, the results will improve further. Although the sample size of the present study and the number of involved PIs and physicians was rather small, we were able to collect two to three cases per physician and PI in order to minimize the between-case variance. Furthermore, the existence of a pool of 12 different PIs trained in physical examination feedback skills is rather unique.

## Conclusions

In summary, our study demonstrates that trained PIs are able to provide feedback of equal quantitative value to that of faculty staff physicians with regard to a physical examination performed on them. However, both the students and the video raters judged the quality of the feedback given by the physicians to be significantly better than that of the PIs. This is interesting insofar as PIs are already trained in providing feedback prior to the specialization in physical examination. Thus, under considerations of personnel and financial resources, it is reasonable to deploy PIs to assess students’ physical examination skills. Further studies should investigate whether these results could be improved further by PIs who have completed longer training or who possess greater experience in the area of physical examination.
